# Special Issue: Research on Herpes Virus Fusion and Entry

**DOI:** 10.3390/v16111788

**Published:** 2024-11-18

**Authors:** Doina Atanasiu, Tina M. Cairns

**Affiliations:** Department of Basic and Translational Sciences, School of Dental Medicine, University of Pennsylvania, Philadelphia, PA 19104, USA

Herpesviridae comprise a large family of enveloped DNA viruses with a unifying ability to establish a latent infection in their host. Though tropism differs, all herpesviruses have the same core fusion machinery that consists of three virus-encoded glycoproteins, gB, and a heterodimer of gH and gL. Studies of herpesviruses in each of the three subfamilies (i.e., alpha, beta, and gamma) have shown that each one embellishes on the core fusion machinery with additional proteins that may serve as receptor-binding proteins or are needed for entry into specific cell types. The solution of the structures of gB and gH/gL of herpes simplex virus (HSV), varicella zoster virus (VZV), Epstein–Barr virus (EBV), human cytomegalovirus (HCMV), and pseudorabies virus (PrV) provides insight into the way these viral glycoproteins execute the fusion of the viral and cellular membranes.

This Special Issue offers five original research papers presenting the latest developments in the contribution of viral glycoproteins gB, gH/gL, and gD from HCMV [1], HSV [2], PrV [3], and KSHV [4] to the infection process, as well as an innovative live-cell plaque reduction assay (PRA) using GFP-expressing HCMV to analyze the impact of antibodies on HCMV spread within fibroblast monolayers [5]. HSV possesses a highly specialized membrane fusion machinery that is vital for infectivity and spread, particularly within neurons. This fusion mechanism, primarily mediated by viral glycoproteins, is essential for viral entry into neuronal axons and the establishment of latency in the cell bodies of neurons. Another review included in this Special Issue, by Jambunathan et al. [6], highlights that, despite the progress in understanding HSV entry mechanisms, many aspects remain unclear, particularly the specific roles of viral and cellular proteins in regulating membrane fusion and intracellular signaling. Unraveling these mechanisms could provide deeper insights into how HSV has evolved to infect neurons stealthily, evading cellular immune responses.

The viral fusogen gB plays a critical role in viral entry and spread. Given its immunodominance, gB has become a key target for vaccine development [7,8]. Reuter et al. [1] found that specific regions of the cytoplasmic tail domain are crucial for maintaining the gB protein in a fusion-inactive state and that interactions with cellular proteins regulate gB-induced fusion in HCMV. This study could inform the design of more effective HCMV vaccines that elicit fusion-inhibiting antibodies and lead to future investigations into the role of gH/gL in modulating gB-mediated fusion during viral infection.

Essential conformational changes in glycoprotein gD are crucial for the viral entry process [9,10]. Receptor binding triggers a significant conformational shift in gD, particularly in the C-terminal domain, exposing the binding sites necessary for recruiting other viral glycoproteins, like gH/gL and gB, to mediate membrane fusion. Atanasiu et al. [2] found a key inflection point in gD that facilitates the necessary structural rearrangements of the C-terminal region for fusion, which affects gD’s ability to bind gH/gL.

While essential for HSV, gD is dispensable for the cell-to-cell spread of PrV [11,12]. Vallbracht et al. [3] shows that compensatory mutations in the N-terminal [13] or transmembrane domains of gH bypass the need for gL to induce gL-independent fusion. The ability of specific mutations to bypass the gL function offers a fascinating glimpse into the intrinsic flexibility of the fusion apparatus, the possible role of gH oligomerization, and the structural contributions of the transmembrane domain. The locations of these mutations also highlight potential new targets for antiviral strategies.

Like other herpesviruses, KSHV relies on the conserved core glycoproteins (gB, gH/gL) for viral entry, with additional glycoproteins that contribute to the virus’s broad cellular tropism. No single receptor is essential across all contexts, suggesting that KSHV employs redundant entry pathways involving multiple receptors and glycoproteins. A key player in KSHV entry into immune cells is DC-SIGN (CD209), a calcium-dependent lectin receptor expressed on dendritic cells, macrophages, monocytes, and B lymphocytes. DC-SIGN facilitates immune cell signaling and adhesion and serves as an entry receptor for multiple viruses, including KSHV. KSHV’s gB glycoprotein binds to DC-SIGN in a dose-dependent manner, although this interaction has not yet been deemed essential for viral entry into any specific cell type [14]. Palmerin et al. [4] provide insightful data on the complex role of DC-SIGN and the KSHV glycoprotein gH in KSHV infection of tonsil-derived B cells. The study suggests that KSHV utilizes two distinct mechanisms of entry into tonsil-derived B cells: a gH-dependent mechanism that does not require DC-SIGN, and a gH-independent mechanism that relies on DC-SIGN.

The editors would like to thank the authors for their scholarly contributions to this Special Issue, and the reviewers for their time and insight. Herpes viruses employ a complex system of entry, utilizing multiple glycoproteins. The studies presented in this Special Issue help to piece together not only how this process works but also which parts of the process to target when developing new antiviral therapies.
